# The Treatment of Exophthalmic Goitre

**Published:** 1904-09-10

**Authors:** 


### THE TREATMENT OF EXOPHTHALMIC GOITRE.

The present-day treatment of exophthalmic goitre is not very satisfactory, and even when followed by improvement, this is usually very slow. Especially is some more promptly acting method to be desired for those cases in which the symptoms soon become acute and death follows in the course of a few days. These facts, and a study of the general phenomena of the disease, suggest the existence of a toxæmic process and the possibility of meeting it by an antitoxic serum. Dr. Geo. R. Murray[1] has proposed that this latter end may be attained by treating an animal with gradually-increasing doses of thyroid extract, in the hope of producing "anti" bodies in the blood, and then using the serum in the treatment of patients suffering from Graves' disease. The animals selected for this purpose were rabbits, and the extract was introduced into the rabbit's mouth by means of a small syringe. It was found that for prolonged administration a 5-minims dose of the extract thrice a week was as much as could be given; under larger or more frequent doses the animals lost weight, and some of them died. The serum was obtained from these rabbits under proper precautions, and was tried in two cases of exophthalmic goitre. In each there were decided symptoms of improvement but not, perhaps, definitely greater than often takes place in such cases under the influence of rest in a hospital ward. Yet Dr. Murray is hopeful that if larger animals than rabbits were employed and larger doses of thyroid were given it might be possible to obtain a serum which would produce decidedly beneficial results. Dr. Robert Hutchison[2] describes two cases of Graves' disease in which he had used a method of treatment proposed by Pitres—namely, the injection into the thyroid gland of a solution of iodoform in ether (1 in 5). This had been used in one case on 19 and in the other on 15 occasions, a dose (1 cubic centimetre) being ordered every two or three days. In each instance there had been a considerable increase in the hardness of the thyroid swelling, a decided fall in the pulse-rate, and a marked improvement in the patient's general condition; the exophthalmos was not much affected. There were no ill-effects. The suggestion is that the injections produce a fibroid change in the thyroid gland and that as a result of this there is a diminution of the excessive secretion upon which, presumably, the symptoms of the disease depend. The method may thus be compared with operative removal of part of the thyroid but without the risks that belong to that procedure.

[1] Lancet, August 27, 1904. [2] Polyclinic, August 1904.

